# Longitudinal multi-trajectory phenotypes of severe eosinophilic asthma on type 2 biologics treatment^☆^

**DOI:** 10.1016/j.waojou.2024.101000

**Published:** 2024-11-21

**Authors:** Duong Duc Pham,, Ji-Hyang Lee,, Hyouk-Soo Kwon,, Woo-Jung Song,, You Sook Cho,, Hyunkyoung Kim,, Jae-Woo Kwon,, So-Young Park,, Sujeong Kim,, Gyu Young Hur,, Byung Keun Kim,, Young-Hee Nam,, Min-Suk Yang,, Mi-Yeong Kim,, Sae-Hoon Kim,, Byung-Jae Lee,, Taehoon Lee,, So Young Park,, Min-Hye Kim,, Young-Joo Cho,, ChanSun Park,, Jae-Woo Jung,, Han Ki Park,, Joo-Hee Kim,, Ji-Yong Moon,, Pankaj Bhavsar, Ian M. Adcock,, Kian Fan Chung,, Tae-Bum Kim,

**Affiliations:** a Department of Allergy and Clinical Immunology, Asan Medical Center, University of Ulsan College of Medicine, Seoul, South Korea; b Department of Allergy and Clinical Immunology, Kangwon National University School of Medicine, Chuncheon, South Korea; c Division of Pulmonary, Allergy and Critical Care Medicine, Chung-Ang University Gwangmyeong Hospital, South Korea; d Department of Internal Medicine, School of Medicine, Kyungpook National University, Daegu, South Korea; e Department of Internal Medicine, Korea University College of Medicine, Seoul, South Korea; f Department of Internal Medicine, Korea University Medical Center Anam Hospital, Seoul, South Korea; g Department of Internal Medicine, Dong-A University College of Medicine, Busan, South Korea; h Department of Internal Medicine, Seoul Metropolitan Government-Seoul National University Boramae Medical Center, Seoul, South Korea; i Department of Internal Medicine, Busan Paik Hospital, Inje University College of Medicine, Busan, South Korea; j Department of Internal Medicine, Division of Allergy and Clinical Immunology, Seoul National University Bundang Hospital, Seongnam, South Korea; k Departments of Medicine, Samsung Medical Center, Sungkyunkwan University School of Medicine, Seoul, South Korea; l Department of Internal Medicine, Ulsan University Hospital, University of Ulsan College of Medicine, Ulsan, South Korea; m Department of Internal Medicine, Eulji University School of Medicine, Seoul, South Korea; n Department of Internal Medicine, Ewha Womans University College of Medicine, Seoul, South Korea; o Department of Allergy and Clinical Immunology, Ewha Womans University Mokdong Hospital, Ewha Womans University College of Medicine, Seoul, South Korea; p Department of Internal Medicine, Inje University Haeundae Paik Hospital, Busan, South Korea; q Department of Internal Medicine, Chung-Ang University College of Medicine, Seoul, South Korea; r Department of Allergy and Clinical Immunology, Kyungpook National University Chilgok Hospital, School of Medicine, Kyungpook National University, Daegu, South Korea; s Department of Internal Medicine, Hallym University Sacred Heart Hospital, Anyang, South Korea; t Department of Internal Medicine, Hanyang University College of Medicine, Seoul, South Korea; u National Heart and Lung Institute, Imperial College London, United Kingdom

**Keywords:** Multi-trajectory analysis, Type 2 biologics, Severe eosinophilic asthma

## Abstract

**Background:**

Limited understanding exists regarding the progression trajectory of severe eosinophilic asthma (SEA) patients on type 2 biologics therapies.

**Objective:**

We aim to explore distinct longitudinal phenotypes of these patients based on crucial asthma biomarkers.

**Methods:**

We enrolled 101 adult patients with SEA. Of these, 51 were treated with anti-IL5/IL5Rα or anti-IL5/IL5RαR antibody, and 50 with anti-IL-4Rα antibody. Multi-trajectory analysis, an extension of univariate group-based trajectory modeling, was used to categorize patients based on their trajectories of forced expiratory volume in 1 s (FEV_1_), blood eosinophil counts (BEC), and fractional exhaled nitric oxide (FeNO) levels at baseline, and after 1, 6, and 12 months of treatment. Associations between trajectory-based clusters and clinical parameters were examined.

**Results:**

Among anti-IL5/IL5Rα antibody-treated patients, 2 clusters were identified. The cluster characterized by higher baseline BEC and lower FEV_1_ showed a better response, with improvements in FEV_1_ and reductions in BEC over time. Among anti-IL-4Rα antibody-treated, 3 clusters were identified. Clusters with moderate BEC and FeNO at baseline demonstrated better improvements in FEV_1_ and reductions in FeNO, despite increased BEC during follow-up. Conversely, individuals with extremely low FeNO and high BEC at baseline were more likely to experience poorer progression, demonstrating an increase in FeNO and a reduction in FEV_1_.

**Conclusion:**

To optimally monitor treatment response in SEA patients on type 2 biologics, integrating longitudinal biomarker features is essential.

## Introduction

Severe asthma (SE) constitutes 5–10% of asthma patients and is associated with poor response to conventional asthma treatments, frequent exacerbations, and irreversible lung function decline.^1^^,^^2^ Molecular analysis of asthma has identified several endotypes, particularly a type 2 (T2) high inflammation phenotype, leading to the development of monoclonal antibody agents (mAbs) targeting severe eosinophilic asthma (SEA).^3^, ^4^, ^5^ In the T2 inflammatory pathway, signals from epithelial cells trigger immune responses, activating effector cells. This activation promotes cytokine production, including *IL-4*, *IL-5*, and *IL-13*, facilitating the recruitment, maturation, migration, and infiltration of eosinophil cells from the bloodstream into the tissue. This process leads to blood eosinophilia and airway eosinophilic inflammation.^6^ Over the past decade, anti-IL5/IL5Rα or anti-IL5/IL5RαR (benralizumab, mepolizumab, and reslizumab), and anti-IL-4Rα (dupilumab) mAbs, have become widely available for treating SEA patients.^3^^,^^7^ Clinical trials consistently demonstrate the significant efficacy of these mAbs in SEA patients, resulting in reduced exacerbation rates, improved lung function, and decreased corticosteroid dependence.^8^ However, individual responses have shown variation and time-dependent alterations.^9^

Asthma, a heterogeneous chronic condition, is characterized by a complex interplay of various pathological factors and their dynamic interactions over time.^10^ Recent studies suggest that the evolution of asthma varies considerably over time. Employing an unsupervised classification approach, Odling et al categorized the trajectory of asthma from infancy to adulthood into 4 groups: infrequent, early-onset or transient, adolescent-onset, and persistent asthma.^11^ Research on the longitudinal trajectory of SEA progression in adulthood is lacking significantly. In a previous study examining 5-year data on lung function, blood eosinophils count (BEC), and inhaled corticosteroid (ICS) usage among adults with mild-to-severe asthma, we identified 6 distinct trajectory-based phenotypes with unique characteristics regarding asthma progression.^12^ Bulow et al. employed severity-related variables to classify 4 SEA trajectories in adults.^13^ However, these studies did not include data from SEA patients using mAbs. Analyzing asthma progression trajectories in SEA patients using mAbs could offer insights into the optimal utilization of these expensive medications.

Variability in eosinophil levels indicates fluctuating airway inflammation over time. Hastie and colleagues discovered that the longitudinal variation in sputum eosinophils correlated with variations in BEC, categorizing them into 3 subgroups: persistent eosinophil high, persistent eosinophil low, and large variability groups.^14^ Fractional exhaled nitric oxide (FeNO) is another non-invasive proxy of T2 airway inflammation. Elevated FeNO levels are associated with a higher exacerbation frequency, with a particularly strong association observed in patients exhibiting high FeNO and high BEC levels.^15^ When treating SEA with mAbs, a high BEC is a crucial criterion for determining the use of anti-IL5/IL5Rα antibody treatment. Meanwhile, when considering anti-IL-4Rα antibody treatment, both BEC and FeNO levels are considered in deciding the treatment.^9^ In SEA patients, lung function serves as an outcome measure to assess the treatment effectiveness. The anti-inflammatory efficacy of mAbs helps improve respiratory function in SEA patients. However, the impact varies among different mAbs and individual patients. We hypothesize that analyzing the trajectories of BEC, FeNO, and lung function simultaneously over time would provide a more comprehensive understanding of SEA progression in mAbs users.

The Precision Medicine Intervention in Severe Asthma (PRISM) study is a multicenter ongoing prospective cohort study involving 27 nationwide centers. We enrolled SEA patients using mAbs with stringent selection criteria and rigorous monitoring.^16^ In this study, we aim to investigate the progression trajectories of SEA patients who are treated with anti-IL5/IL5Rα and anti-IL-4Rα mAbs as part of the PRISM study. Additionally, our focus is on key biomarker indicators, including BEC, FeNO, and lung function.

## Methods

### Participants

**Fig. 1:**
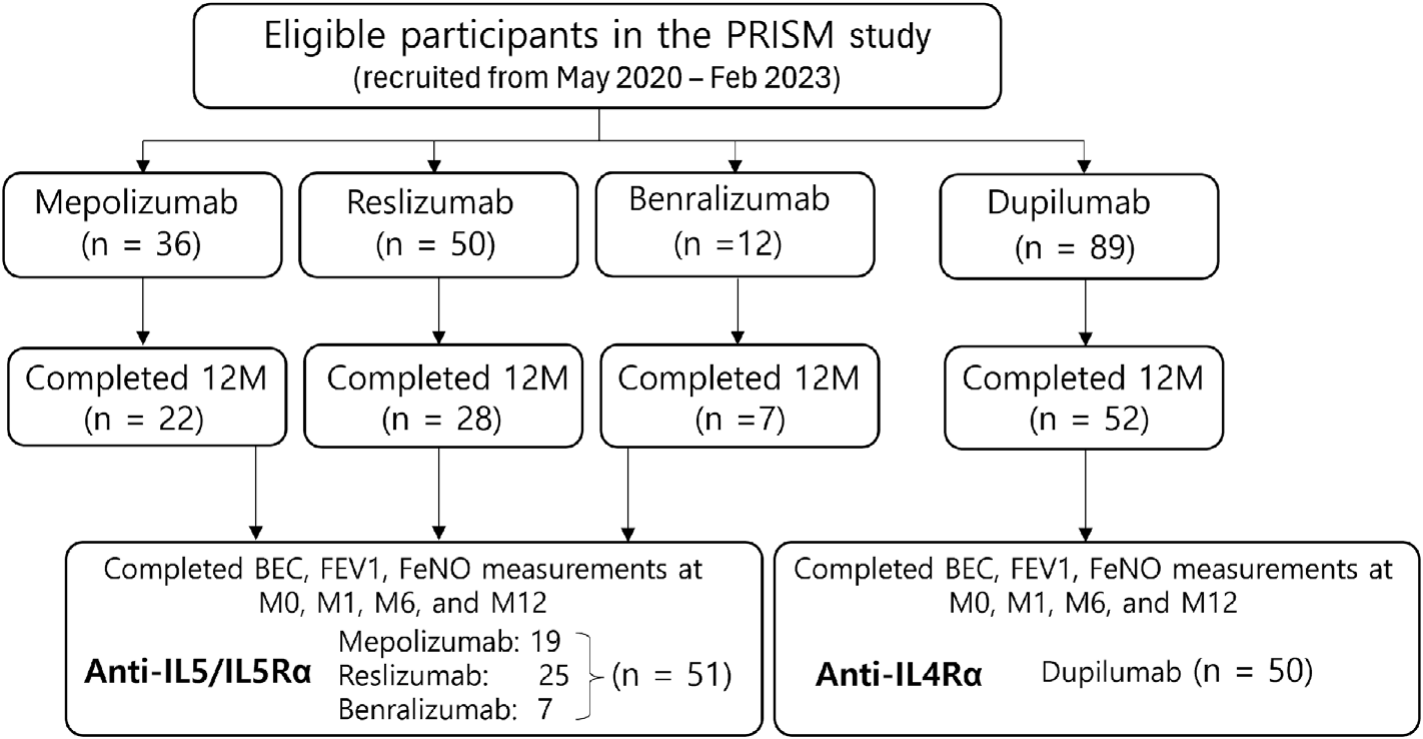
Study flow

In the PRISM study, patients with SA aged over 18 years were enrolled. SA was identified by a respiratory expert based on guidelines of the European Respiratory Society (ERS) and American Thoracic Society (ATS).^17^ All patients in the PRISM study were either "controlled" while on high-dose continuous ICS or a second anti-asthma drug, or "uncontrolled" despite active treatment. Patients eligible for biologics as an add-on therapy should not have used any biologics for asthma treatment in the past 3 months, should not have had prolonged use of OCS in the past 4 weeks (more than a total of 90 mg prednisolone), should not have hypereosinophilic syndrome, allergic bronchopulmonary aspergillosis, eosinophilic granulomatosis with polyangiitis, or any other severe respiratory diseases. The biologics used in the PRISM study included anti-IgE (omalizumab), anti-IL5/IL5Rα (benralizumab, mepolizumab, and reslizumab), and anti-IL-4Rα (dupilumab).^16^ After administering biologics, patients were monitored at 4 data points: baseline, 1, 6, and 12 months. In this study, we exclusively included patients treated with anti-IL5/IL5Rα or anti-IL-4Rα mAbs who underwent a minimum twelve-month follow-up. Throughout the year, lung function, BEC, and FeNO measurements were taken at 4 data points. In total, 51 patients received anti-IL5/IL5Rα (19 on mepolizumab, 25 on reslizumab, and 7 on benralizumab), and 50 patients received anti-IL-4Rα mAbs (dupilumab), as depicted in Fig. 1. The research protocol received approval from the Institutional Review Board at each study site and was registered on ClinicalTrials.gov (registration no. NCT05164939).^18^ All participants provided informed written consent.

### Biologics treatment

In the PRISM study, mepolizumab (Nucala, GlaxoSmithKline, NC, USA) was administrated to patients with a baseline BEC of ≥150 cells/μL or ≥300 cells/μL in at least 1 test within the previous 12 months. It was administered every 4 weeks as a 100 mg subcutaneous injection. Reslizumab (Cinqair, Teva-Handok, Seoul, Korea) was indicated for patients with a BEC >400 cells/μL, with a dose of 3 mg/kg intravenously every 4 weeks. Benralizumab (Fasenra, AstraZeneca Korea Co, Seoul, Korea) was administered subcutaneously at 30 mg every 4 weeks for the first 3 doses, followed by every 8 weeks thereafter, to SEA patients with a baseline BEC higher than 150 cells/μL. Dupilumab was administered as a 300 mg subcutaneous injection every 2 weeks (Dupixent, Sanofi-Aventis, Seoul, Korea). It was indicated for patients with a baseline BEC >150 cells/μL or FeNO >25 ppb. Each month, dupilumab users received the first dose at the hospital and an additional take-home dose for self-administration under strict supervision.

### Measurements

The longitudinal variables utilized in multi-trajectory analysis include prebronchodilator FEV_1_% predicted values, BEC, and FeNO. Spirometry was performed by a trained technician following a standard operating procedure. The BEC values, measured in cells per μL, were calculated from standard complete blood cell counts. FeNO measurements were conducted using an airway inflammation monitor (Niox Vero, SE-754, Uppsala, Sweden) following ATS/ERS guidelines and technical standards.^19^ All patients in the multi-trajectory analysis had complete data points for FEV_1_, BEC, and FeNO measured at baseline, as well as after 1, 6, and 12 months.

Other longitudinal variables, including ICS (in budesonide equivalent dose – Bud_eq) and OCS (in prednisolone equivalent dose – Pred_eq) use, self-reported asthma control test (ACT) questionnaire results, the proportion of high-dose ICS use (Bud_eq > 400 mg/d − ICS_high_) and maintenance of OCS (OCS_main_), were collected. Detailed instructions for calculating the steroid equivalent dose are available in eTable 1. Additional baseline characteristics, such as age of diagnosis (Age_dia_), age of starting asthma treatment (Age_treat_), the number of recall exacerbations during the previous year, sputum eosinophils (Sp_EOS) measured as percentages obtained from an induced sputum sample after the patient had inhaled hypertonic saline, and the history of comorbidities, including allergic rhinitis (AR), gastric reflux (GR), and nasal polyps (NPs), were also collected.

### Statistical analysis

Statistical analyses were conducted using R version 4.3.1 and statistical significance was set at *P* < 0.05. Before performing trajectory analysis, BEC values were log-transformed (BEC_log_). To assign patients based on their trajectories of FEV_1_, BEC_log_, and FeNO, we utilized multi-trajectory analysis (MTA), an extension of the univariate group-based trajectory modeling (GBTM) pioneered by Nagin and colleagues.^20^, ^21^, ^22^ GBTM utilizes finite mixture modeling with polynomial regression to identify distinct groups of individuals exhibiting similar trajectory patterns. MTA was conducted by estimating the probability of linking membership in trajectory groups across different predictors. We performed MTA with a varying number of clusters, ranging from 1 to 10. Then, we selected the model with the lowest Bayesian information criterion (BIC) as the optimal choice. The number of trajectory-based clusters was determined based on the optimal cluster count, the proportion of individuals within each cluster, and clinical relevance. To evaluate the baseline characteristics across trajectory-based clusters, analysis of variance was applied to continuous variables exhibiting a normal distribution. The rank sum test was applied for continuous variables demonstrating a non-normal distribution, while Fisher's exact test was employed for categorical variables. A linear mixed-effects model, adjusted for age and sex, was employed to compare the baseline characteristics and the slope of change over 12 months for FEV_1_, BEC, FeNO, Bud_eq, Pred_eq, and ACT scores. The mean exacerbation during 12-month follow-up across trajectory-based clusters was compared using the rank sum test. To compare the annual exacerbation rates of the trajectory-based clusters over 1 year, a negative binomial (NB) model was utilized, adjusting for age and sex. Logistic regression analysis and sensitivity–specificity equality criterion (SSEC)^23^ were employed to access the predictive power and determine the optimal cutoff for baseline characteristic covariates to distinguish between better and poorer response clusters. All analyses were performed separately for anti-IL5/IL5Rα and anti-IL-4Rα mAbs.

## Results

### Anti-IL5/IL5Rα treatment

**Fig. 2:**
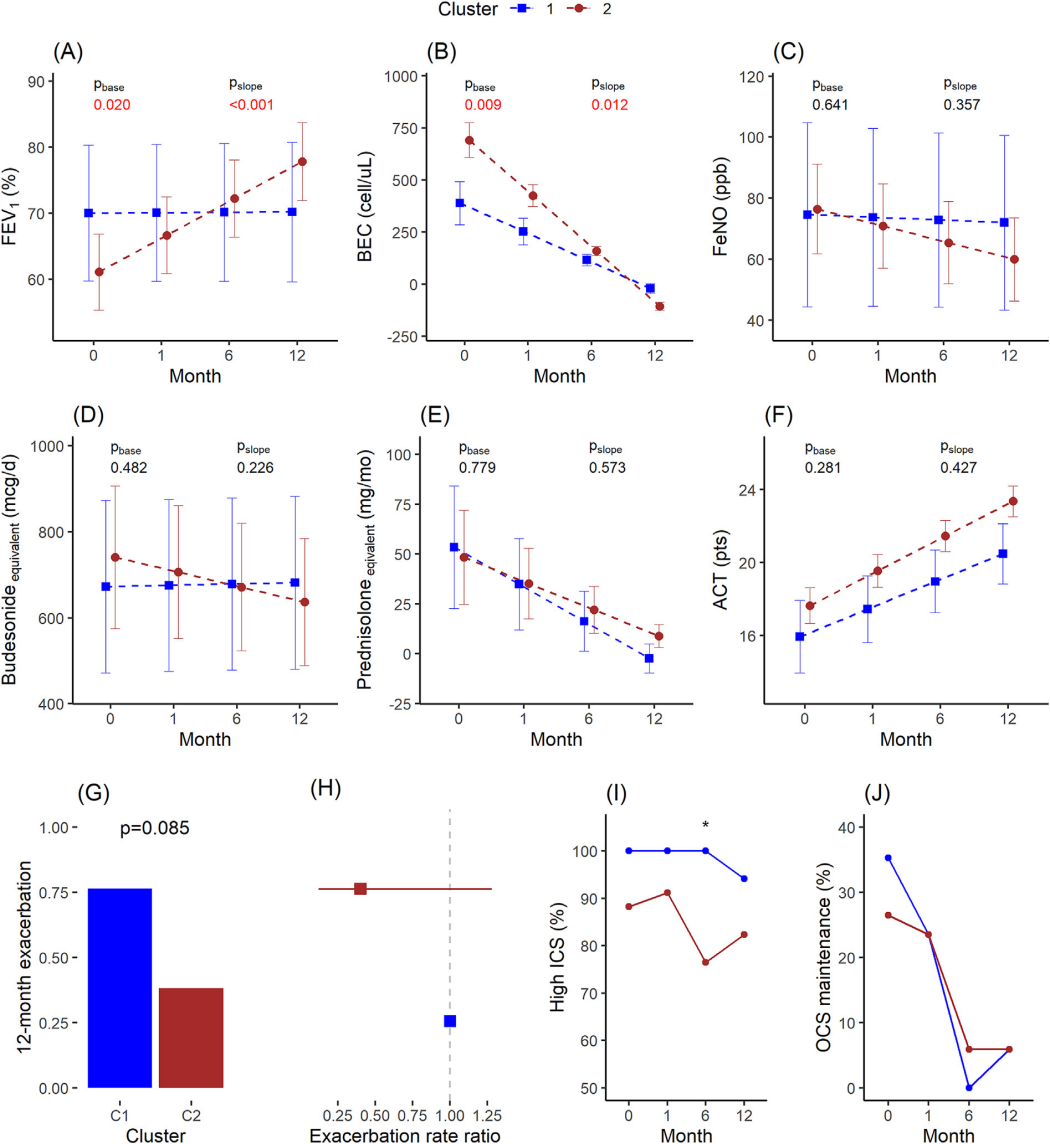
Clinical parameters during 12-month follow-up across trajectory-based clusters among anti-IL5/IL5Rα antibody-treated patients. (A) Predicted FEV_1_ (%), (B) blood eosinophil counts, (C) FeNO, (D) budesonide equivalent (mcg per day), (E) prednisolone equivalent dose (mg per month), and (F) asthma control test score. The data are presented as mean with 95% coefficient intervals estimated by linear mix-effect regression, adjusted for age and sex. P_base_ and P_slope_ are p-values for group differences in baseline levels and the slope of change, respectively. Significant *P-values* are indicated in red. (G) Mean exacerbation events during the 12-month follow-up period. *P*-values were estimated using the rank-sum test. (H) The exacerbation rate ratio was estimated using negative binomial regression, adjusted for age and sex, with Cluster 1 as the reference. (I) Proportion of high-dose ICS users. (J) Proportion of OCS maintenance. *P*-values were estimated using the Fisher exact test. ∗, *P*-value <0.05

The MTA revealed that the optimal number of multi-trajectory-based clusters for users of anti-IL5/IL5Rα mAbs was determined to be 2. (eTable 2). Cluster 1, comprising 33% of cases, exhibited a significantly lower baseline BEC compared to that of Cluster 2 (*P* = 0.001) (Table 1). Additionally, Cluster 2 displayed a higher FEV_1_ and lower Spu_EOS compared to that of Cluster 2, although this difference did not reach statistical significance (*P* = 0.07). Other factors, including demographics, exacerbation history, steroid use, and comorbidity, did not differ between clusters (Table 1). The mixed-effect linear analysis, adjusted for age and sex, revealed that although the baseline FEV1 of Cluster 2 was lower (*P* = 0.02), it showed a substantial improvement over 12 months, while the FEV_1_ of Cluster 1 remained stable (Fig. 2A). At baseline, the BEC of Cluster 2 was higher (*P* = 0.009), and the rate of decrease in BEC over time for Cluster 2 was also higher (*P* = 0.012) than that of Cluster 1 (Fig. 2B). There were no differences observed in the baseline and slope of change regarding FeNO, Bud_eq, Pred_eq, and ACT between the 2 trajectory-based clusters (Fig. 2C, D, E, and F). The average exacerbation rate over the 12-month follow-up period in Cluster 1 was approximately twice that of Cluster 2 (*P* = 0.085) (Fig. 2G). However, there was no difference in the adjusted exacerbation rate between the 2 groups (Fig. 2H). In Cluster 1, all patients used high-dose ICS and continued this treatment after 6 months; however, this proportion slightly decreased after 12 months. In contrast, Cluster 2 started with a lower proportion of high-dose ICS usage, which decreased over time. The proportion of high-dose ICS users in Cluster 2 was lower than that in Cluster 1 at the 6-month treatment mark (*P* = 0.044) (Fig. 2I). Both clusters exhibited a decrease in the proportion of patients requiring maintenance OCS over time (Fig. 2J).

**Table 1:** Baseline characteristics of trajectory-based clusters among anti-IL5/IL5Rα antibody-treated patients.

	C1	C2	p
n (%)	17 (33.3)	34 (66.7)	
Male n (%)	9 (52.9)	10 (29.4)	0.131
Age (yrs)	50.9 (11.4)	53.4 (10.8)	0.458
Age_dia (yrs)	37.6 (15.0)	41.2 (13.9)	0.274
Age_treat (yrs)	39.7 (14.2)	41.8 (13.8)	0.412
BMI (kg/m^2^)	25.1 (4.3)	24.0 (3.1)	0.351
% FEV_1_ (%)	70.3 (22.0)	58.8 (17.6)	0.071
BEC (cell/μL)	528.4 (460.7)	937.1 (524.3)	**0.001**
Spu_EOS (%)	20.9 (32.2)	31.0 (29.9)	0.075
FeNO (ppb)	77.1 (70.6)	75.4 (50.7)	0.646
ACT (pts)	14.9 (6.0)	16.7 (4.6)	0.284
Last year EXA (n)	1 (5.9)	3 (8.8)	0.662
Bud_eq (mcg/d)	675.3 (399.0)	730.3 (506.7)	0.901
High_ICS n (%)	17 (100.0)	30 (88.2)	0.288
Pred_eq (mg/mo)	59.4 (102.5)	38.6 (77.4)	0.367
Main OCS n (%)	6 (35.3)	9 (26.5)	0.532
Allergic rhinitis n (%)	12 (70.6)	25 (73.5)	0.999
Gastric reflux n (%)	5 (29.4)	8 (23.5)	0.738
Nasal polyp n (%)	4 (23.5)	7 (20.6)	0.999

Data are presented as mean (SD) or n (%). C1–C2, 2 multi-trajectory-based clusters. N (%), the number (%) of patients assigned. *P-values* were determined using Fisher's exact test for categorical variables, ANOVA for continuous variables, and the rank sum test for non-parametric data. *P-values* < 0.05 are indicated in bold. Age_diag_, the age at asthma diagnosis; Age_treat_, the age at initiation of asthma treatment; BEC, blood eosinophil count; Spu_EOS, sputum eosinophils as a percentage; %FEV_1_, predicted pre-bronchodilator forced expiratory volume in the first second; Bude_eq, budesonide equivalent; Pred_eq, prednisolone equivalent; ACT, asthma control test score; EXA, exacerbation; High_ICS, Bud_eq higher than 400 mcg/d; mainOCS, maintenance OCS

In terms of lung function improvement and BEC reduction, Cluster 2 demonstrated a superior response to anti-IL5/IL5Rα mAbs medication. Baseline levels of BEC and FEV_1_ exhibited the highest predictive power for identifying Cluster 2. To predict Cluster 2, 1 standard deviation (SD) increase in BEC yielded an odds ratio (OR) of 3.24 (1.40−10.01), while for FEV1, it was 0.52 (0.25−0.98). BEC levels above 600 cells/μL corresponded to an AUC of 0.80, with sensitivity and specificity levels of approximately 0.80 (eTable 3).

### Anti-IL-4Rα treatment

**Fig. 3:**
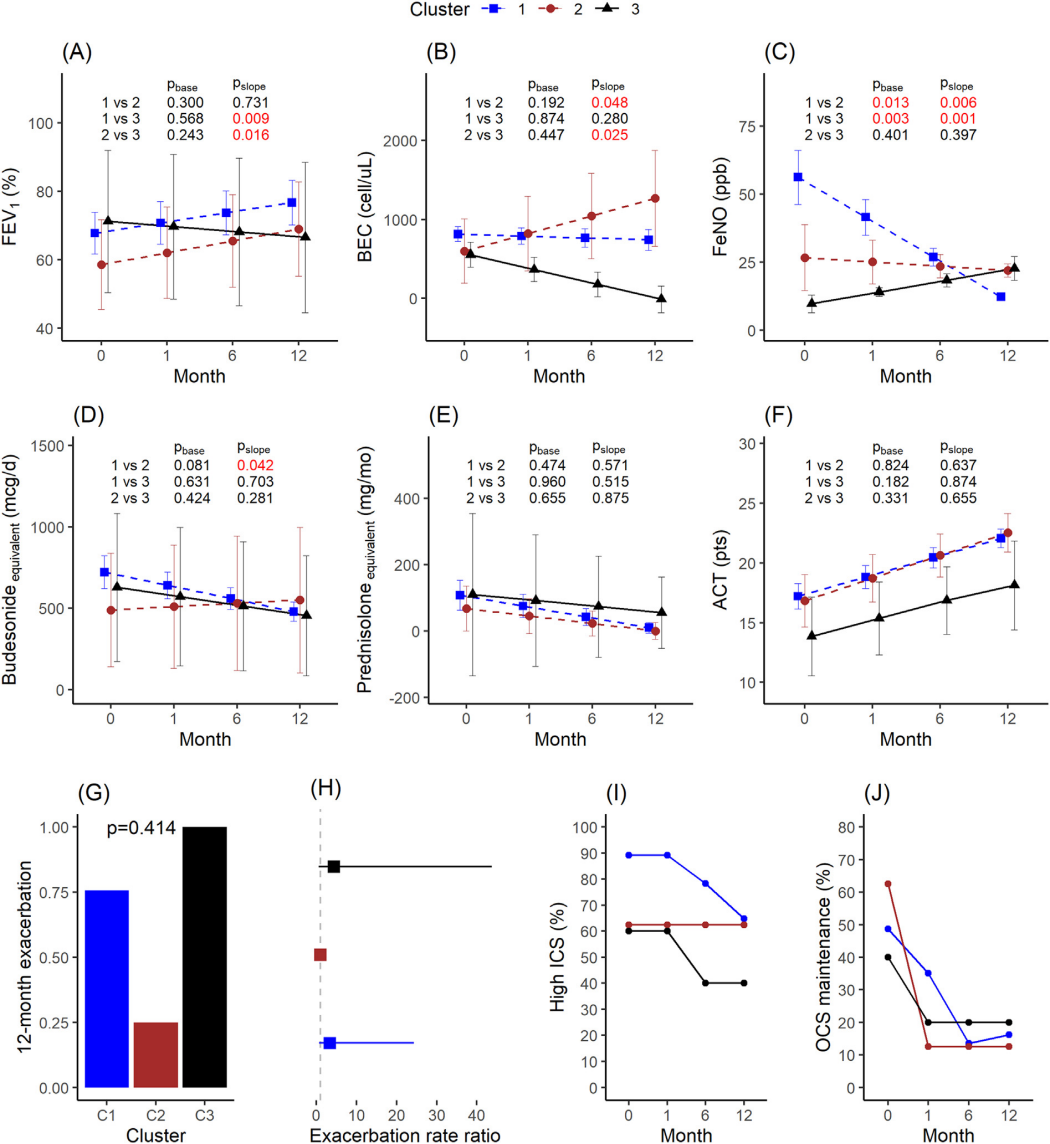
Clinical parameters during 12-month follow-up across trajectory-based clusters among anti-IL-4Rα antibody-treated patients. (A) Predicted FEV_1_ (%), (B) blood eosinophil counts, (C) FeNO, (D) budesonide equivalent (mcg per day), (E) prednisolone equivalent dose (mg per month), and (F) asthma control test score. The data are presented as mean with 95% coefficient intervals estimated by linear mix-effect regression, adjusted for age and sex. P_base_ and P_slope_ are p-values for group differences in baseline levels and the slope of change, respectively. Significant *P-values* are indicated in red. (G) Mean exacerbation events during the 12-month follow-up period. *P*-values were estimated using the rank-sum test. (H) The exacerbation rate ratio was estimated using negative binominal regression, adjusted for age and sex, with Cluster 2 as the reference. (I) Proportion of high-dose ICS users. (J) Proportion of OCS maintenance. *P*-values were estimated using the Fisher exact test. ∗, *P*-value <0.05

The MTA revealed that the optimal number of multi-trajectory-based clusters for anti-IL-4Rα mAb-treated patients was determined to be 3 (eTable 4). At baseline, Cluster 1 displayed higher levels of both BEC and FeNO. Cluster 3 exhibited the highest BEC levels but the lowest FeNO levels, whereas Cluster 2 showed the lowest BEC levels but medium FeNO levels (Table 2). During the 12-month follow-up period, Clusters 1 and 2 demonstrated an improvement in FEV_1_, whereas Cluster 3 experienced a decline in FEV_1_ (all *P-values* for the slope of change <0.05) (Fig. 3A). Over the 12 months of medication use, BEC increased steadily in Cluster 2 but decreased gradually in Clusters 1 and 3 (all *P-values* for the slope of change <0.05), with the most notable decline observed in Cluster 3 (Fig. 3B). The trends in FeNO fluctuations among the clusters diverged from those in BEC fluctuations. Cluster 1 exhibited the highest FeNO levels at baseline (all *P-values* baseline <0.05) and showed the most significant decrease (all *P-values* slope <0.01) after medication use. In contrast, Cluster 3 had lower FeNO levels at baseline and experienced a slight increase after medication use (Fig. 3C). There were no significant differences in the longitudinal variation of Bud_eq, Pred_eq, and ACT scores across clusters (Fig. 3D, E, and F). The mean number of exacerbation events was lowest in Cluster 2 and highest in Cluster 3, although this difference was not statistically significant (Fig. 3G). The adjusted exacerbation rate ratios of Clusters 1 and 3 were 3.36 (0.72–24.3) and 4.36 (0.58–43.8) respectively, compared to Cluster 2. Cluster 2 maintained the proportion of high-dose ICS users, while the other clusters showed a slight reduction in this proportion (Fig. 3H). All clusters exhibited a decrease in the proportion of patients requiring maintenance OCS over time (Fig. 3J). However, no statistically significant differences were found in the proportion of high-dose ICS and OCS maintenance use across the clusters over time.

**Table 2:** Baseline characteristics of trajectory-based clusters among anti-IL-4Rα antibody-treated patients.

	C1	C2	C3	p
n (%)	37 (74)	8 (16)	5 (10)	
Male n (%)	18 (48.6)	5 (62.5)	1 (20.0)	0.453
Age (yrs)	54.2 (8.8)	54.8 (10.0)	55.2 (11.9)	0.967
Age_dia (yrs)	41.1 (11.8)	39.9 (10.7)	36.0 (12.6)	0.708
Age_treat (yrs)	41.9 (12.0)	41.4 (11.6)	36.5 (12.2)	0.687
BMI (kg/m^2^)	25.0 (3.9)	26.1 (2.3)	25.1 (3.1)	0.717
% FEV_1_ (%)	64.2 (19.5)	56.2 (18.0)	68.4 (18.3)	0.475
BEC (cell/μL)	636.0 (549.2)	237.6 (215.4)	749.4 (1311.6)	**0.011**
Spu_EOS (%)	19.2 (22.9)	27.6 (38.5)	3.8 (4.0)	0.531
FeNO (ppb)	65.3 (43.7)	26.1 (15.4)	10.4 (3.4)	**<0.001**
ACT (pts)	16.8 (5.0)	15.2 (5.3)	14.6 (2.1)	0.523
Last year EXA (n)	3 (8.1)	0 (0.0)	0 (0.0)	0.88
Bude_eq (mcg/d)	683.9 (377.3)	455.0 (291.7)	608.8 (373.7)	0.121
High_ICS n (%)	33 (89.2)	5 (62.5)	3 (60.0)	0.076
Pred_eq (mg/mo)	118.2 (175.1)	81.2 (101.6)	89.9 (134.1)	0.911
Main OCS n (%)	18 (48.6)	5 (62.5)	2 (40.0)	0.797
Allergic rhinitis n (%)	27 (73.0)	5 (62.5)	4 (80.0)	0.872
Gastric reflux n (%)	17 (45.9)	2 (25.0)	4 (80.0)	0.213
Nasal polyp n (%)	6 (16.2)	0 (0.0)	1 (20.0)	0.505

Data are presented as mean (SD) or n (%). C1–C3, 3 multi-trajectory-based clusters. N (%), the number (%) of patients assigned. *P-values* were determined using Fisher's exact test for categorical variables, ANOVA for continuous variables, and the rank sum test for non-parametric data. *P-values* < 0.05 are indicated in bold. For other abbreviations, see Table 1

In terms of lung function improvement and reduction in FeNO levels, Cluster 2 demonstrated a superior response, while Cluster 3 showed a poorer response (Table 2). Baseline BEC and FeNO levels showed the highest predictive power for identifying Cluster 2 (Table 2). To predict Cluster 2, an increase of 1 SD in BEC and FeNO corresponded to OR and 95% CI of 0.04 (0.00−0.50) and 0.17 (0.02–0.73), respectively. BEC levels below 305 cells/μL and FeNO levels below 40 ppb were associated with an AUC of 0.78 and 0.75, respectively, demonstrating high sensitivity and specificity levels (eTable 5). Baseline FeNO level showed the highest predictive power for predicting Cluster 3. FeNO levels below 15 ppb had an AUC of 0.97 with high sensitivity and specificity levels (eTable 6).

## Discussion

Investigating the progression of asthma-related parameters can offer valuable insights into the diverse development of asthma, particularly in SEA patients. However, traditional univariate trajectory modeling approaches do not consider the simultaneous interplay of clinical factors predicting asthma response to mAbs. Herein, a novel approach called MTA was applied to investigate asthma progression phenotypes using longitudinal data from 3 key asthma-related parameters: FEV_1_, BEC, and FeNO, in SEA patients treated with available type 2 biologics therapies.

Distinct trajectory-based clusters, exhibiting differences in baseline characteristics and progression patterns of clinical parameters, were identified among users of anti-IL5/IL5Rα and anti-IL-4Rα mAb treatments. Among anti-IL5/IL5Rα mAb-treated patients, FEV_1_ and BEC primarily shaped the trajectory pattern, while FeNO played an additional and independent role in the trajectory pattern of anti-IL-4Rα mAb-treated patients. Within the anti-IL5/IL5Rα group, 2 clusters were identified. A cluster characterized by higher baseline BEC and lower FEV_1_ levels exhibited more favorable progression, showing improvements in FEV_1_ and reductions in BEC over time. Among anti-IL-4Rα mAb-treated patients, 3 clusters were discerned. Clusters with moderate baseline levels of both BEC and FeNO showed greater improvements in FEV1 and decreases in FeNO over time, despite an increase in BEC during follow-up. Conversely, individuals with extremely low FeNO and high BEC levels at baseline were more likely to experience poorer progression, exhibiting an increase in FeNO and a reduction in FEV_1_.

BEC is a non-invasive and cost-effective proxy for eosinophilia, a key feature of T2 inflammation in SEA patients. The increase in blood eosinophils promotes their infiltration into the airway, leading to inflammation.^24^ Mepolizumab and reslizumab directly inhibit *IL5*, reducing the production and maturation of eosinophils in the bone marrow, whereas benralizumab blocks the IL5-Rα receptor, inducing apoptosis in peripheral eosinophils.^6^ All of these anti-IL5/IL5Rα mAb-treatments immediately and effectively lower BEC levels, and this reduction persists over time.^25^, ^26^, ^27^ In contrast, the anti-IL-4Rα mAb (dupilumab) does not affect eosinophil production and growth. Instead, it impedes these cells from adhering to blood vessels and exiting circulation, thereby preventing them from reaching target tissues and reducing localized eosinophilic inflammation, such as in the respiratory tract.^3^^,^^6^^,^^28^ Consequently, there is a temporary rapid increase in BEC after administering anti-IL-4Rα mAb, followed by a gradual reduction.^29^^,^^30^ Thus, BEC progression should be monitored differently in those treated with anti-IL5/IL5Rα and anti-IL-4Rα mAbs. In this study, among anti-IL5/IL5Rα mAb-treated patients, the degree of BEC reduction correlated with the improvement in lung function and, to some extent, with the severity of exacerbations during the follow-up period. However, this was not observed in the group treated with anti-IL-4Rα. Cluster 3 participants, despite experiencing a persistent decrease in BEC, exhibited poor progression characterized by decreased lung function. Conversely, Cluster 2 participants, experiencing an increase in BEC, showed significant improvement.

The key distinction between Clusters 2 and 3 in patients using anti-IL-4Rα mAb relied on the dynamic FeNO profiles. Cluster 2 exhibited a declining trend in FeNO levels, whereas Cluster 3 showed a progressive increase in FeNO. BEC is considered reflective of effector cell activity and circulating *IL5* levels, whereas FeNO is a direct indicator of T2 airway inflammation, regulated by the *IL13*-related pathway.^31^ Although this may suggest that, for patients using anti-IL-4Rα mAb, monitoring FeNO is more important than BEC for identifying good responders. However, it's essential to note that poor responders among Cluster 1 patients exhibited a significant decrease in FeNO but no temporal variation in BEC. Monitoring the response to anti-IL-4Rα treatment should consider both BEC and FeNO dynamics. Given anti-IL-4Rα mAb's inhibition of eosinophil adhesion to vascular endothelium and tissue emigration,^3^^,^^6^^,^^28^ a short-term increase in BEC may be more notable than a decrease or unchanged response. Among patients treated with anti-IL5/IL5Rα mAb, the difference in FeNO dynamic profiles between the 2 groups was less evident, although clusters with good progression exhibited a slight decrease in FeNO over time.

The contrasting roles of BEC and FeNO in predicting the response to mAbs also indicate the distinct contributions of these markers to disease progression among those treated with anti-IL5/IL5Rα versus anti-IL-4Rα mAbs. SEA patients treated with anti-IL5/IL5Rα, especially those with very high BEC levels before treatment, particularly ≥600 cells/μL, are more likely to be classified in the good responder group (Cluster 2), consistent with previous studies.^27^^,^^32^^,^^33^ In contrast, the baseline FeNO levels showed no correlation with trajectory-based clusters in anti-IL5/IL5Rα mAb-treated patients. In the PRISM study, the criteria for administering anti-IL-4Rα were SEA patients with high BEC or high FeNO, leading to the inclusion of some patients with very high BEC but low FeNO levels. These patients were likely to belong to the poor responder group (Cluster 3). High baseline FeNO is associated with an increased risk of future exacerbations^15^ and the response to anti-IL-4Rα mAb therapy.^34^ However, in our study, the baseline FeNO appeared to exhibit a U-shaped relationship with the response to anti-IL-4Rα mAb therapy. Extremely low baseline FeNO levels were associated with Cluster 3, the poorest response group, while extremely high baseline FeNO levels corresponded to Cluster 1, a group that showed improved lung function but also experienced high exacerbation rates. The ideal baseline FeNO range appeared to be above 15 ppb but below 40. These findings need to be validated in another study with a larger sample size.

Our findings should be interpreted in the context of the study's strengths and limitations. This is the first study, to our knowledge, to utilize trajectory analysis based on integrated information from 3 key clinical features in the response of SEA patients to different type 2-directed mAbs. Monitoring the dynamics of multiple influencing factors over time simultaneously provides a more comprehensive view of the heterogeneity of disease progression in asthma. This real-life study more accurately reflects the complexity of clinical settings. However, its limitations, such as the regression to the mean phenomenon, should be considered when interpreting the results. Because this study utilized a single-arm pre-post biologic design, we were unable to account for a potential placebo effect. The data also emphasizes the independence of FeNO and BEC in defining T2 airway inflammation and the response to mAb therapy. However, because this study only followed patients for 12 months, the long-term variations of the variables were not observed. It has been demonstrated that lung function in severe asthma patients gradually and irreversibly declines over time.^35^ In the anti-IL-4Rα-treated patient group, BEC exhibited a short-term increase and then decreased after 12 months.^29^^,^^30^ Similar multi-trajectory analyses should be conducted on SEA patient groups with longer follow-up periods. The sample size affects the number of classes that can be detected in GBTM analysis, with larger sample sizes increasing the likelihood of detecting additional classes.^36^ Monte Carlo simulation shows that the number of groups seems to reach a saturation point at a sample size of about 200^36^^,^^37^. In this study, each biologics group contained only about 50 subjects. Due to the relatively small sample size, the estimates may be unstable, and the trend clusters may not fully represent the larger population. Furthermore, due to the small sample size of each subgroup within the anti-IL5/IL5Rα mAbs group, we were unable to conduct separate analyses for each medication. The mechanisms underlying the effects of treatment with anti-IL5/IL5Rα mAbs involve a direct inhibition of IL5 and the IL5-Rα receptor, which are distinct. These differences could lead to variations in drug responses and trajectory changes over time.

In conclusion, MTA presents a novel approach to evaluating the disease progression patterns in SEA patients treated with type 2 biologics therapies, particularly in contexts where this progression necessitates simultaneous consideration of multiple factors. Clinical parameters in SEA patients vary significantly among different patient groups and with each type of mAbs used. Trajectory-based clustering may assist in predicting patient responses to mAbs and enhance drug selection for SEA patients.

## Availability of data and materials

The data-sets analyzed during the current study are available from the corresponding author on reasonable request.

## Authors’ contributions

DDP and TBK designed the study; JHL, HSK, WJS, YSC, HK, JWK, SYP, SK, GYH, BKK, YHN, MSY, MYK, SHK, BJL, TL, SYP, MHK, YJC, CP, JWL, HKP, JHK, and JYM collected the data; DDP analyzed the data; DDP and TK interpreted the data; DDP drafted the manuscript; PK, IA, KFC revised the manuscript critically for important intellectual content; all authors critically reviewed and approved the manuscript as submitted and agreed to be accountable for all aspects of the work.

## Ethics approval and consent to participate

The research protocol received approval from the Institutional Review Board at each study site and was registered on ClinicalTrials.gov (registration no. NCT05164939). All participants provided informed written consent.

## Authors’ consent for publication

All authors agreed to the publication of this work in the World Allergy Organization Journal.

## Funding

This research was supported by the Bio & Medical 10.13039/100006180Technology Development Program of the 10.13039/501100001321National Research Foundation funded by the Korean government [2019M3E5D3073365].

## Declaration of competing interest

The authors have no conflicts of interest to declare.
