# Supplementary material for: Longitudinal multi-trajectory phenotypes of severe eosinophilic asthma on type 2 biologics treatment
Source: World Allergy Organ J. 2024 Nov 21;17(12):101000. doi: 10.1016/j.waojou.2024.101000 (PMC11617764; doi:10.1016/j.waojou.2024.101000)
Supplement: Multimedia component 1 [file mmc1.docx]

**eTable 1. Conversion of the usage and dosage of inhaled and oral corticosteroid**

| Budesonide equivalent dose | | | |  | Prednisolone equivalent dose | | |
| --- | --- | --- | --- | --- | --- | --- | --- |
| Generic compound | Brand name | Total/day | coefficient |  | Generic compound | Total/month | coefficient |
| Fluticasone/salmeterol | Seretide, Fluterol, Airflusal Forspiro | ...mcg | X 2 |  | Prednisolone | ...mg | X 1 |
| Budesonide/formoterol | Symbicort, Duorespi Spiromax | ...mcg | X 1 |  | Methylprednisolone | ...mg | X 1.25 |
| Beclomethasone/formoterol | Foster | ...mcg | X 1 |  | Calcort | ...mg | X 0.83 |
| Fluticasone/formoterol | Flutiform | ...mcg | X 2 |  | Dexamethasone | ...mg | X 6.67 |
| Fluticasone/vilanterol | Relvar Ellipta | ...mcg | X 1 |  | Hydrocortisone | ...mg | X 0.25 |
| Ciclesonide | Alvesco | ...mcg | X 2.5 |  | Triamcinolone | ...mg | X 1.25 |
| Fluticasone propionate | Flixotide | ...mcg | X 2 |  |  |  |  |
| Fluticasone furoate | Arnuity | ...mcg | X 1 |  |  |  |  |
| Budesonide | Pulmicort, Obucor, Prenorine, Budiair, Infulammide | ...mcg | X 1 |  |  |  |  |
| Beclomethasone | Becobent, Beclomet easyhaler | ...mcg | X 2 |  |  |  |  |

**eTable2. Optimal cluster number for anti-IL5/IL5Rα antibody-treated patients**

| Cluster | 2 clusters | 3 clusters | 4 clusters | 5 clusters | 6 clusters | 7 clusters | 8 clusters | 9 clusters | 10 clusters |
| --- | --- | --- | --- | --- | --- | --- | --- | --- | --- |
| 1 | 17 (33.3%) | 22 (43.1%) | 26 (51%) | 18 (35.3%) | 2 (3.9%) | 3 (5.9%) | 3 (5.9%) | 3 (5.9%) | 3 (5.9%) |
| 2 | 34 (66.7%) | 15 (29.4%) | 7 (13.7%) | 7 (13.7%) | 7 (13.7%) | 1 (2%) | 1 (2%) | 6 (11.8%) | 5 (9.8%) |
| 3 |  | 14 (27.5%) | 15 (29.4%) | 17 (33.3%) | 17 (33.3%) | 7 (13.7%) | 7 (13.7%) | 7 (13.7%) | 5 (9.8%) |
| 4 |  |  | 3 (5.9%) | 6 (11.8%) | 3 (5.9%) | 17 (33.3%) | 8 (15.7%) | 7 (13.7%) | 7 (13.7%) |
| 5 |  |  |  | 3 (5.9%) | 19 (37.3%) | 2 (3.9%) | 9 (17.6%) | 9 (17.6%) | 9 (17.6%) |
| 6 |  |  |  |  | 3 (5.9%) | 18 (35.3%) | 2 (3.9%) | 1 (2%) | 1 (2%) |
| 7 |  |  |  |  |  | 3 (5.9%) | 18 (35.3%) | 4 (7.8%) | 4 (7.8%) |
| 8 |  |  |  |  |  |  | 3 (5.9%) | 11 (21.6%) | 3 (5.9%) |
| 9 |  |  |  |  |  |  |  | 3 (5.9%) | 11 (21.6%) |
| 10 |  |  |  |  |  |  |  |  | 3 (5.9%) |
| BIC | 1224 | 1234 | 1267 | 1342 | 1342 | 1245 | 1273 | 1340 | 1387 |

Data are presented as the number and % of patients assigned by multi-trajectory modeling. BIC, Bayesian information criterion (the lowest BIC indicates the best model)

**eTable3. The descriptive power of baseline characteristics in predicting the better response cluster (C2) in anti-IL5/IL5Rα antibody-treated patients**

| Predictor | OR | AUC | Cutoff | Se | Sp |
| --- | --- | --- | --- | --- | --- |
| BEC* | **3.24 (1.4−10.01)** | 0.8 | >=600 | 0.79 | 0.82 |
| FEV_1_* | **0.52 (0.25−0.98)** | 0.66 | <=65 | 0.68 | 0.65 |
| Sex | 0.37 (0.11**−**1.23) | 0.62 | Male | 0.71 | 0.53 |
| Age_diag | 1.3 (0.72**−**2.47) | 0.6 | >=39 | 0.59 | 0.59 |
| ACT* | 1.44 (0.8**−**2.69) | 0.59 | >=17 | 0.56 | 0.59 |
| Age | 1.27 (0.7**−**2.41) | 0.58 | >=52 | 0.56 | 0.59 |
| Age_treat | 1.17 (0.64**−**2.18) | 0.57 | >=43 | 0.56 | 0.53 |
| High_ICS | 0 (0**−**0) | 0.56 | YES | 0.12 | 0.99 |
| BMI* | 0.73 (0.39**−**1.31) | 0.56 | <=25 | 0.59 | 0.47 |
| Prednisolone_eq* | 0.79 (0.44**−**1.42) | 0.56 | <=30 | 0.76 | 0.29 |
| OCS_maintenance | 0.66 (0.19**−**2.38) | 0.54 | YES | 0.74 | 0.35 |
| FeNO* | 0.97 (0.54**−**1.83) | 0.54 | >=58 | 0.56 | 0.59 |
| His_EXA | 0.82 (0.45**−**1.48) | 0.54 | <=2 | 0.65 | 0.47 |
| Gastric_Reflux | 0.74 (0.2**−**2.88) | 0.53 | YES | 0.76 | 0.29 |
| Allergy_Rhinitis | 1.16 (0.3**−**4.15) | 0.51 | YES | 0.74 | 0.29 |
| Nasal_polyp | 0.84 (0.21**−**3.69) | 0.51 | YES | 0.79 | 0.24 |
| Budesonide_eq* | 1.13 (0.63**−**2.26) | 0.49 | <=640 | 0.71 | 0.24 |

*, OR for one SD increase; AUC, area under the curve of ROC; Se, sensitivity; Sp, specificity **eTable4. Optimal cluster number for anti-IL-4Rα antibody-treated patients**

| Cluster | 2 clusters | 3 clusters | 4 clusters | 5 clusters | 6 clusters | 7 clusters | 8 clusters | 9 clusters | 10 clusters |
| --- | --- | --- | --- | --- | --- | --- | --- | --- | --- |
| 1 | 45 (90%) | 37 (74%) | 7 (14%) | 7 (14%) | 7 (14%) | 8 (16%) | 8 (16%) | 7 (14%) | 7 (14%) |
| 2 | 5 (10%) | 8 (16%) | 32 (64%) | 26 (52%) | 17 (34%) | 17 (34%) | 11 (22%) | 11 (22%) | 11 (22%) |
| 3 |  | 5 (10%) | 6 (12%) | 6 (12%) | 5 (10%) | 5 (10%) | 5 (10%) | 5 (10%) | 5 (10%) |
| 4 |  |  | 5 (10%) | 5 (10%) | 6 (12%) | 4 (8%) | 3 (6%) | 3 (6%) | 3 (6%) |
| 5 |  |  |  | 6 (12%) | 5 (10%) | 5 (10%) | 7 (14%) | 6 (12%) | 6 (12%) |
| 6 |  |  |  |  | 10 (20%) | 2 (4%) | 5 (10%) | 5 (10%) | 2 (4%) |
| 7 |  |  |  |  |  | 9 (18%) | 2 (4%) | 2 (4%) | 2 (4%) |
| 8 |  |  |  |  |  |  | 9 (18%) | 6 (12%) | 6 (12%) |
| 9 |  |  |  |  |  |  |  | 5 (10%) | 3 (6%) |
| 10 |  |  |  |  |  |  |  |  | 5 (10%) |
| BIC | 1430 | 1420 | 1427 | 1468 | 1543 | 1555 | 1622 | 1662 | 1692 |

Data are presented as the number and % of patients assigned by multi-trajectory modeling. BIC, Bayesian information criterion (the lowest BIC indicates the best model)

**eTable5. Descriptive power of baseline characteristics in predicting the better response cluster (C2) among anti-IL-4Rα antibody-treated patients.**

| Predictor | OR | AUC | Cutoff | Se | Sp |
| --- | --- | --- | --- | --- | --- |
| BEC* | **0.04 (0−0.5)** | 0.78 | <=305 | 0.75 | 0.71 |
| FeNO* | **0.17 (0.02−0.73)** | 0.75 | <=40 | 0.75 | 0.67 |
| Budesonide_eq* | 0.35 (0.08**−**1.05) | 0.72 | <=620 | 0.75 | 0.64 |
| BMI* | 1.36 (0.64**−**2.83) | 0.64 | >=25 | 0.62 | 0.64 |
| FEV_1_* | 0.65 (0.3**−**1.38) | 0.64 | <=61 | 0.75 | 0.62 |
| Gastric_Reflux | 0.33 (0.05**−**1.64) | 0.62 | YES | 0.75 | 0.50 |
| High_ICS | 0.28 (0.05**−**1.63) | 0.62 | YES | 0.38 | 0.86 |
| Sex | 2.02 (0.44**−**10.88) | 0.59 | Male | 0.62 | 0.55 |
| Nasal_polyp | 0 (0**−**0) | 0.58 | YES | 0.99 | 0.17 |
| ACT* | 0.77 (0.34**−**1.66) | 0.58 | <=16 | 0.62 | 0.55 |
| OCS_maintenance | 1.83 (0.4**−**9.88) | 0.57 | YES | 0.62 | 0.52 |
| His_EXA | 1.25 (0.57**−**2.35) | 0.57 | <=1 | 0.62 | 0.50 |
| Allergy_Rhinitis | 0.59 (0.12**−**3.26) | 0.56 | YES | 0.38 | 0.74 |
| Age | 1.05 (0.49**−**2.4) | 0.53 | <=56 | 0.62 | 0.43 |
| Prednisolone_eq* | 0.78 (0.25**−**1.68) | 0.53 | <=30 | 0.62 | 0.45 |
| Age_treat | 1 (0.47**−**2.25) | 0.50 | <=42 | 0.50 | 0.44 |
| Age_diag | 0.94 (0.43**−**2.08) | 0.48 | <=40 | 0.50 | 0.46 |

*, OR for one SD increase; AUC, area under the curve of ROC; Se, sensitivity; Sp, specificity

**eTable6. The descriptive power of baseline characteristics in predicting the poorer response cluster (C3) in anti-IL-4Rα antibody-treated patients**

| Predictor | OR | AUC | Cutoff | Se | Sp |
| --- | --- | --- | --- | --- | --- |
| FeNO* | **0 (0−0.01)** | 0.97 | <=15 | 0.99 | 0.89 |
| Gastric_Reflux | 5.47 (0.74**−**111.74) | 0.69 | YES | 0.8 | 0.58 |
| BEC* | 1.26 (0.47**−**2.51) | 0.69 | <=320 | 0.8 | 0.64 |
| Sex | 0.24 (0.01**−**1.77) | 0.66 | Male | 0.8 | 0.51 |
| ACT* | 0.67 (0.24**−**1.71) | 0.64 | <=16 | 0.8 | 0.56 |
| Age_treat | 0.64 (0.21**−**1.81) | 0.63 | <=42 | 0.75 | 0.47 |
| High_ICS | 0.28 (0.04**−**2.38) | 0.62 | YES | 0.4 | 0.84 |
| Age_diag | 0.65 (0.22**−**1.85) | 0.62 | <=40 | 0.5 | 0.47 |
| Age | 1.11 (0.44**−**3.17) | 0.58 | >=57 | 0.6 | 0.6 |
| FEV_1_* | 1.39 (0.54**−**4.33) | 0.57 | >=70 | 0.6 | 0.6 |
| OCS_maintenance | 0.64 (0.08**−**4.2) | 0.56 | YES | 0.6 | 0.51 |
| Prednisolone_eq* | 0.86 (0.21**−**2.04) | 0.55 | <=5 | 0.6 | 0.49 |
| Allergy_Rhinitis | 1.62 (0.21**−**33.4) | 0.54 | YES | 0.8 | 0.29 |
| His_EXA | 1.02 (0.27**−**2.17) | 0.54 | <=2 | 0.6 | 0.38 |
| Budesonide_eq* | 0.9 (0.26**−**2.1) | 0.54 | <=640 | 0.8 | 0.24 |
| Nasal_polyp | 1.62 (0.08**−**13.62) | 0.53 | YES | 0.2 | 0.87 |
| BMI* | 0.97 (0.33**−**2.34) | 0.48 | >=25 | 0.6 | 0.62 |

*, OR for one SD increase; AUC, area under the curve of ROC; Se, sensitivity; Sp, specificity
